# Performance evaluation of the compounding robot, APOTECAchemo, for injectable anticancer drugs in a Japanese hospital

**DOI:** 10.1186/s40780-017-0081-z

**Published:** 2017-04-24

**Authors:** Takuya Iwamoto, Takuya Morikawa, Miki Hioki, Hirofumi Sudo, Demis Paolucci, Masahiro Okuda

**Affiliations:** 1 Department of Pharmacy, Mie University Hospital, 2-174 Edobashi, Tsu, Mie 514-8507 Japan; 2 Loccioni Humancare, Moie di Maiolati, Ancona, Italy

**Keywords:** Robotic preparation, Chemotherapy, Wipe test, APOTECAchemo, Japanese hospital, Pharmacy automation

## Abstract

**Background:**

The accuracy, safety and feasibility of, the compounding robot APOTECAchemo were evaluated in the clinical practice of Japan.

**Methods:**

Accuracy and precision of robotic preparations by APOTECAchemo was evaluated in 20 preparations of fluorouracil (FU) and cyclophosphamide (CPA) infusions by four pharmacists. Environmental and product contaminations with FU and CPA were evaluated by wipe testing. Robotic performance was compared with manual preparation in a biological safety cabinet. The number of robotic products, total compounding time and total pre-reconstitution time of lyophilized drugs between January 1, 2014 to December 31, 2015 were investigated.

**Results:**

Robotic preparation resulted more accurate and precise (mean absolute dose error and coefficient of variation were 0.83 and 1.04% for FU and 0.52 and 0.59% for CPA) than those of manual preparation (respective values were 1.20 and 1.46% for FU and 1.70 and 2.20% for CPA). Drug residue was not detected from any of the prepared infusion bags with the robotic preparation, whereas FU was detected in two of four analyzed infusion bags with manual preparation. Average total time to make single anticancer drug preparation (compounding plus reconstitution of lyophilized drugs) was 6.11 min in the second half of 2015. During the study period, the highest percentage of production covered by APOTECAchemo was 70.4% of the total inpatient pharmacy activity.

**Conclusion:**

Robotic preparation using APOTECAchemo should give substantial advantages in drug compounding for accuracy and safety and was able to be successfully worked in Mie university hospital.

## Background

The preparation of anticancer drugs has become an increasing complex matter of concern due to the continuous approval of innovative drugs for more highly personalized therapy, which raises the possibility of compounding error [1–4]. As well the risk of exposure of patients and oncology workers to carcinogens during handling is a persistent hazard [5–8]. In Japan, pharmacists are the main professional in charge of preparation of anticancer agents. The recent enlarging scope of professional activities of the pharmacist in oncology and hematology area, such as adjusting medication, ordering, interpreting and monitoring laboratory tests, developing therapeutic plans and educating patients, creates difficulty in time-consuming and complex procedure for the safe preparation of anticancer drugs [9, 10]. Therefore, hospital pharmacists currently make a difficult challenge in designing an accurate and efficient preparation method while considering the prevention of exposure to anticancer drugs for healthcare workers.

As a consequence, robotic devices have attracted much attention and have gradually spread all over the world. At present, the compounding robot APOTECAchemo (Loccioni Humancare, Italy) has been used in 51 hospitals in 14 countries [11, 12]. Robotic preparation leads to solve the problem of manual preparation, such as exposure or contact of hazardous drugs, stress of preparation, human error of preparation, and weakness of traceability for preparation error. The Japanese experience with robotics in pharmacy are described in two publications that evaluated the robotics use for injectable anticancer drugs in each clinical setting [13, 14]. One report described the reduced workplace contamination measured by surface wipe testing after introducing robotics (CytoCare; TOSHO Co., Ltd, Tokyo), whereas only 9 liquid drugs (20% of total preparations) were applicable for robotic preparation [13]. The other publication described the reduction of the use of closed system drug transfer devices after introduction of robotics (ChemoRo; Yuyama Co., Ltd, Osaka) [14]. However, this preparation robot took a much longer time compared to manual preparation.

APOTECAchemo was introduced for the first time in Japan to Mie University Hospital in July 2012. In this study, we evaluate the performance of APOTECAchemo in terms of dose accuracy, environmental and product contamination with hazardous drugs, and preparation time, to assess its feasibility for Japanese clinical practice.

## Methods

### Accuracy and precision of robotic preparations

A 2-week study was performed from November 11 to November 25, 2012 to compare the dose accuracy between the robotic preparations and the traditional manual compounding. The drug selected were Fluorouracil (FU) and Cyclophosphamide (CPA) as respectively the most commonly used liquid and lyophilized drugs. Twenty preparations of 800 mg of FU in 500 mL Normal Saline (NS) bag and twenty of 400 mg of CPA in 100 mL NS bag were prepared with both methodologies. The effective amount of active ingredient dosed is measured gravimetrically by weighing the bags before and after drug injection. Then the volume is calculated by dividing the dosed drug weight for the drug specific gravity. The preparations were equally distributed among 4 different pharmacists. Their months of experience for anticancer drug preparation were 35, 21, 15, and 8 months, respectively.

Dose accuracy and precision were calculated by the mean absolute preparation error (% discrepancy between the compounded and the prescribed drug quantity) and the associated standard deviation. CPA and FU used in this study were Endoxan® (Shionogi & Co., Ltd., Osaka), and 5-FU® (Kyowa Hakko Kirin Company, Limited, Tokyo), respectively.

### Verification of environmental contamination with anticancer drugs

An assessment on the possible contamination of the working areas and the final products was performed on both the robotic and manual procedures. The sampling locations were selected based on risk considerations and five spots were chosen for sampling in the APOTECAchemo robot (loading area where initial drug vials and final products are temporarily placed; surface beneath the shelf where the partially used vials are stocked inside the compounding area, surface beneath the dosing device where the compounding takes place; the scale; the gripper of robot arm used to hold the drugs and disposables). One area was also sampled in the biological safety cabinet (BSC) (surface of the compounding area). In addition, we measured the residual contamination on gloves and final infusion bags after compounding.

Surface wipe sampling for FU and CPA was performed with the wipe test kit produced by Kobelco Research Institute, Inc. These kits contained standardized supplies for taking samples, including certified drug-free sampling tissues, dropper bottles containing sampling solution, storage containers, latex gloves, and labels. Frozen wipe samples were transported with dry ice to Kobelco Research Institute for determination of FU and CPA levels. The analytical methodology used was HPLC-MS/MS system (HPLC: Agilent 1100, Agilent technologies, Tokyo; MS/MS: API4000, AB Sciex, Tokyo). The chromatographic separation was performed at 40 °C on an extended C-18 column. The mobile phase consisted of a combination of phase A (0.1% formic acid with 2 mM ammonium formate) and phase B (acetonitrile). Mass analysis was performed using ESI positive mode. The limit of quantification (LoQ) of the methodology was 0.02 ng/mL.

### Drug stability evaluation of lyophilized drugs after reconstitution

We designed and carried out a stability study on the most used lyophilized drugs in order to evaluate the possibility to reconstitute it in advance. Doxorubicin (DXR) (Adriacin® 50 mg; Kyowa Hakko Kirin Company, Limited, Tokyo), Ifosfamide (IFO) (Ifomide® 1 g; Shionogi & Co., Ltd., Osaka), Gemcitabine (GEM) (Gemcitabine®　1 g; Yakult Honsha Company, Limited, Tokyo) and CPA (Endoxan® 500 mg; Shionogi & Co., Ltd., Osaka) were mixed with NS solution (respectively 10, 25, 25 and 25 mL) and mixed to complete dissolution. A defined volume of each drug (25 μL of IFO and GEM, 50 μL of CPA and 500 μL DXR) was sampled and then diluted with pure water to 10 mL. The final concentration of IFO, GEM and CPA samples was 100 ng/mL, and that of DXR was 208.3 ng/mL. We prepared 5 samples for each drug. The prepared Day 0 samples were used for the measurement of corresponding drug concentrations by HPLC-MS/MS system and those values were set as benchmarks. After preparation, each vial was sealed with sterile tape and stored at 2 °C in the refrigerator. The stored vials were taken out of the refrigerator at 3 pm on Day 1, Day 3, Day 4, Day 5, Day 7 and Day 10 and left at room temperature for 1 h. Sampling and quantitative analysis were performed for each solution following the above mentioned procedure to determine the remaining drug quantity. Finally, the percentages of the remaining active ingredient of the lyophilized drugs were plotted as a function of time after reconstitution.

### Production performance of APOTECAchemo

APOTECAchemo went live in February 26, 2013, in the inpatient pharmacy. We analyzed the production during a 2-year period (from January 1, 2014 to December 31, 2015), after an approximate one-year pilot and ‘on-boarding’ phase using the APOTECAchemo robot. We evaluated the trend of the robotic production, the compounding time as well as the pre-reconstitution (reconstitution was done in advance according to the stability of lyophilized drugs after dissolution. For lyophilized drug preparation, the compounding time includes the reconstitution phase. Typical day running status of APOTECAchemo, composed of compounding time, pre-reconstitution time, and cleaning time was also analyzed for week 46 (Nov 9–13) of 2015.

### Statistical analysis

The statistical analysis was performed using Prism 5 for windows ver. 5.01. The parametric Student’s *t* test or Weltch’s *t* test was performed to investigate the difference of two different samples.

## Results

### Accuracy and precision of robotic preparations

**Fig. 1:**
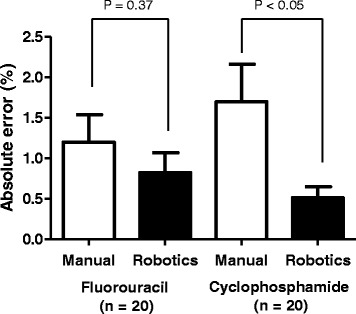
Comparison of percent absolute dose error between manual and robotic compounding of FU and CPA. The statistical analyses were performed using Student’s *t* test or Weltch’s *t* test. Mean and standard error were shown. FU: Fluorouracil; CPA: Cyclophosphamide

Dose accuracy (mean absolute dose error) and precision (coefficient of variation) of robotic of anticancer drugs were 0.83 and 1.04% for FU and 0.52 and 0.59% for CPA, respectively (Fig. 1). In the manual preparation, these values were 1.20 and 1.46% for FU and 1.70 and 2.20% for CPA, respectively. The absolute dose error of the robotic preparation for CPA was significantly smaller than that of manual preparation (*P* < 0.05).

### Verification of environmental contamination with anticancer drugs

Concentrations of CPA and FU in wipe samples from APOTECAchemo and BSC locations are shown in Table 1.

**Table 1:** Concentrations of cyclophosphamide and fluorouracil in wipe samples from APOTECAchemo and biological safety cabinet locations

	Place	Sampling timing	FU (ng/cm^2^)	CPA (ng/cm^2^)
APOTECA chemo (Robotics)	Loading area	Before compounding	nd	nd
		After compounding	nd	nd
	Compounding area, under shelf	Before compounding After compounding	nd nd	nd nd
	Compounding area, under dosing device	Before compounding After compounding	nd nd	Nd 25
	Scale balance	Before compounding	nd	nd
		After compounding	nd	nd
	Gripper of robot arm	Before compounding	nd	nd
		After compounding	nd	0.04
	Gloves 1^st^	After FU compounding	nd	-
	Gloves 2^nd^	After compounding	nd	nd
	Bag 1	After compounding	nd	nd
	Bag 2	After compounding	nd	nd
	Bag 3	After compounding	nd	nd
	Bag 4	After compounding	nd	nd
BSC (Manual)	Inside BSC	Before compounding	nd	nd
		After compounding	12.5	nd
	Gloves 1^st^	After FU compounding	nd	-
	Gloves 2^nd^	After compounding	nd	nd
	Bag 1	After compounding	nd	nd
	Bag 2	After compounding	nd	nd
	Bag 3	After compounding	0.15	nd
	Bag 4	After compounding	1.03	nd

*FU* Fluorouracil, *CPA* Cyclophosphamide, *BSC* biological safety cabinet

nd means < 0.05 ng/cm^2^ for Fluorouracil; < 0.02 ng/cm^2^ for Cyclophosphamide

In APOTECAchemo, CPA concentration over the LoQ was detected after compounding in two locations, the compounding area under the dosing device (25 ng/cm^2^) and the gripper of robot arm (0.04 ng/cm^2^). In the BSC, FU at the concentration of 12.5 ng/cm^2^ was detected.

Although there were two locations where CPA was found in APOTECAchemo, anticancer drug residues were not detected from any of the four tested infusion bags. On the other hand, during manual preparation in BSC, two of four infusion bags had detectable FU at concentrations of 0.15 and 1.03 ng/cm^2^, respectively.

No traces of the contaminants were detected in the gloves of the operators after working with the robot or after the manual compounding.

### Drug stability evaluation measurement of lyophilized drugs after reconstitution

**Fig. 2:**
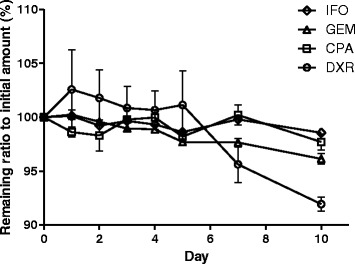
Percent remaining of active ingredient for 4 lyophilized drugs after reconstitution at the recommended concentration for preparation (*n* = 5). Error bar indicates standard error. IFO: (*diamond*); GEM: (*triangle*); CPA: Cyclophosphamide (*square*); DXR: (*circle*)

Figure 2 shows the percent of the remaining active ingredient for 4 lyophilized drugs after reconstitution at each recommended concentration for preparation. The four anticancer drugs investigated, IFO, GEM, CPA and DXR, showed a remaining quantity ratio compared to the initial amount greater than 95.6% during initial 7 days. On Day10, the remaining ratio of DXR was under 95.0%, and the corresponding value was 92.0.

### Production performance of APOTECAchemo

From February 26, 2013, we began to use APOTECAchemo for inpatient preparations. The anticancer drugs handled by robotic preparation (34 drugs, 56 vial size) is shown in Table 2. During the study period 10,217 doses were prepared by the robot and the highest percentage of robotic preparation among total preparation for inpatients was 70.4%.

**Table 2:** Anticancer drugs handled by APOTECAchemo

Ingredient	Content (mg)	Ingredient	Content (mg)
Amrubicin Hydrochloride	20, 50	Ifosfamide	1000
Bendamustine Hydrochloride	100	Irinotecan Hydrochloride Hydrate	40, 100
Bevacizumab (Genetical Recombination)	100, 400	L-Asparaginase	5000 U
Carboplatin	50, 150, 450	Methotrexate	50, 200, 1000
Cetuximab (Genetical Recombination)	100	Oxaliplatin	50, 100, 200
Cisplatin	10, 25, 50	Paclitaxel	30, 100
Cyclophosphamide Hydrate	100, 500	nab-Paclitaxel (abraxane)	100
Cytarabine	1000	Panitumumab (Genetical Recombination)	100
Dacarbazine	100	Pemetrexed Sodium Hydrate	100, 500
Daunorubicin Hydrochloride	20	Pertuzumab (Genetical Recombination)	420
Docetaxel	20, 80	Pirarubicin	10, 20
Doxorubicin Hydrochloride	10, 50	Ramucirumab	100
Doxorubicin Hydrochloride	20	Rituximab (Genetical Recombination)	100, 500
Epirubicin Hydrochloride	10, 50	Temozolomide	100
Etoposide	100	Trastuzumab (Genetical Recombination)	150
Fluorouracil	250, 1000	Vincristine Sulfate	1
Gemcitabine Hydrochloride	200, 1000	Vinorelbine Ditartrate	10, 40

**Fig. 3:**
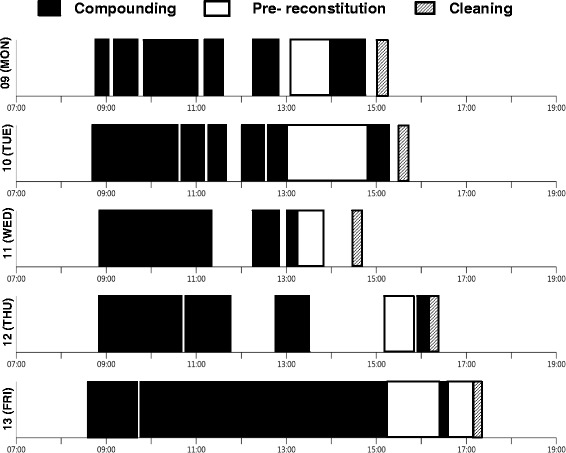
Running status of APOTECAchemo from Nov 9 to Nov 13 2015. Compounding time (*black*), time of pre- reconstitution time of lyophilized drugs (*white*) and cleaning time (*shaded*) were shown

Figure 3 shows the typical day running status of APOTECAchemo. The compounding is mainly concentrated in the morning, approximately from 8.45 am to 11.45 am. Few other preparations are processed in the afternoon, hence we used the spare time to reconstitute in advance the lyophilized drugs that have shown longer stability in the above previous study. At the end of the day, the cleaning of the system requires 15 min. Friday represents an exception with longer production time in the afternoon due to the necessity to prepare the medications planned for the weekend.

**Fig. 4:**
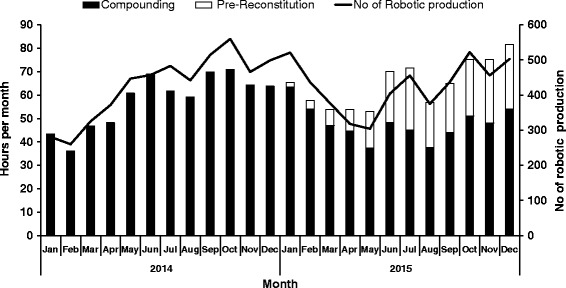
No of robotic production (*solid line*) and total compounding time (*black bar*) and total pre- reconstitution time of lyophilized drugs (*white bar*) per month

The changes in the number of robotic production, total compounding time and total pre-constitution time of lyophilized drugs during the study period are shown in Fig. 4. Initially, the production of the robot progressively increased due to several implementations customized for Japan (e.g., the handling of the Terumo® syringes). This growing trend inverted in early 2015 because of the pharmacy staff rotation and subsequent training of new employees. After June 2015, the production number by robotics gradually rose up to more than 500 medication doses/month. As shown in Fig. 4, we started reconstituting lyophilized drugs in advance (pre-reconstitution) in January 2015 and then this practice boosted in June 2015 once we had the confirmation of reconstituted drug stability study. This led to save time for compounding medication doses, as outlined by the shortening of compounding time for the similar number of medication doses after introducing the pre-reconstitution (black bar in Fig. 4).

**Fig. 5:**
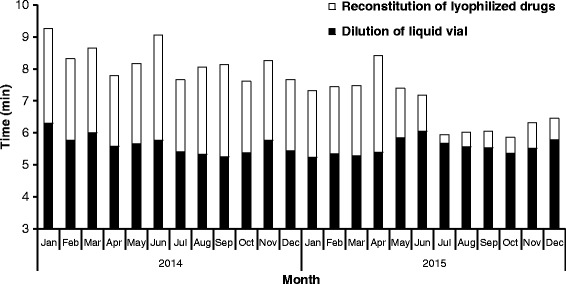
Average of reconstitution time of lyophilized drugs (*white bar*) and average dilution time with liquid vial in the robot (*black bar*) per month

The average reconstitution time of lyophilized drugs (inadequate for pre-reconstitution) and dilution time with ready-to-use vials (liquid drugs and pre-reconstituted lyophilized drugs) in the robot per month are shown in Fig. 5. At the end of 2015, average time of a preparation with a ready-to-use drug was 5.57 min. On the other side, the average preparation time with a lyophilized drug (compounding plus reconstitution) was 6.11 min.

## Discussion

Accurate drug preparation is one of the most essential issues for chemotherapy. In the present study, we showed that robotic preparation using APOTECAchemo had greater accuracy and precision compared to manual preparation. Especially, absolute dose error of CPA in robotic preparation was significantly smaller than that in manual preparation. Meanwhile, although CV% for both robotic and manual preparations were less than 2.5%, robotic preparation was more precise than manual preparation. The high repeatability of the robotic practice, which exactly performs identically programmed maneuvers during each compounding procedure. From January to August 2015, the median rate of absolute dose error more than 5% was 0.7% (range: 0.0–1.9%) for each month in the robotic preparation (total of 3,192 preparations for 34 anticancer drugs) in practice. There is one report comparing the performance between robotic preparation using APOTECAchemo and manual preparation, showing that both preparations were accurate and precise [12]. The range of percent dose error (accuracy) and standard deviation (precision) for robotic preparation were from −3.71 to 0.42% and from 0.57 to 1.92%, respectively. This report also showed that the deviation of percent dose error for robotic preparation indicated negative values, with the exception of cisplatin preparation (0.42%). Our result had a similar tendency, mean preparation error of FU and CPA preparations were −0.68 and −0.41%, respectively (data not shown). In the robotic preparation, the sampling volume of ingredient is decided and measured by the actual added weight of syringe from empty syringe. Therefore, a little bit remaining in the syringe seems to be a main reason for the negative value of the deviation of percent dose error for robotic preparation. As a whole, APOTECAchemo has good accuracy and precision for compounding anticancer drugs, and the risk of overdose preparation must be low as compared to manual preparation, resulting in safe management of cancer treatment.

It is important to evaluate environmental and product contamination when robotic preparation system is newly introduced in the clinical setting [15]. Recently, a wipe test investigated environmental and product contaminations by CPA in the robotic preparation with APOTECAchemo and manual preparation was published [16]. This report showed that the contamination of CPA was observed on the operator’s gloves (4 of 7: 0.0004-0.0967 ng/cm^2^) and the majority (70%) of infusion bags during manual preparation. Instead, during robotic preparation by APOTECAchemo, gloves (1 of 8: 0.0007 ng/cm^2^) and infusion bags (15%) were considerably less contaminated. Our result has also showed that FU was detected in two infusion bags (50%) during manual preparation, whereas anticancer drugs were not detected from any of infusion bags during robotic preparation using APOTECAchemo. These results reasonably suggest that contamination of infusion bags was much lower by using APOTECAchemo as compared with manual preparation. In the robotic preparation, infusion bags never contact the surface of compounding bottom panel where sometimes contaminated by anticancer drugs. This fact was one of the conceivable reason for the small risk of infusion bag contamination in the robotic preparation.

One of the most common concerns to robotic preparation seems to be a prolonged preparation time compared with manual preparation. Long preparation time may restrict the opportunity of robotic preparation in Japan [13]. Indeed, the reported cover rate (the rate of robotic preparations among all anticancer preparations) of robotic preparation using CytoCare® was 23.5% in routine practice, and robotic preparation was used for 9 liquid drugs, paclitaxel, carboplatin, FU, etoposide, irinotecan, oxaliplatin, cisplatin, DXR and cytarabine [13]. The reported average cycle time for single preparation for corresponding drugs by CytoCare was 6.9 min in practice. This data was restricted for liquid drug preparation, however, the way to measure the single preparation time seems to be similar to our study. In another institution, robotic preparation using ChemoRo® was at least applicable for 17 drugs with mean preparation time between 9.1 min (Carboplatin) to 24.0 min (CPA) for single preparation, and cover rate of robotic preparation was about 30% in routine practice [14]. There were two reports regarding the clinical use of APOTECAchemo, and total 47 and 31 anticancer drugs were handled by the robot in Cleveland Clinic in the United States [11], and Wake Forest Baptist Medical Center in the United States [17], respectively. In the latter institution, the reported average cycle time for single preparation by APOTECAchemo was 5.98 min during 27 weeks in 2015 [17]. In our hospital, 56 vial sizes of 34 anticancer drugs were validated for the use in APOTECAchemo, and average cycle time for a single anticancer preparation was 6.11 min in the second half of 2015. The experimental condition differed from each institution, and simple comparison and interpretation regarding preparation time may be inappropriate, however, our results were comparable to those of above the two hospitals in the United States, and the speedy preparation enables us to increase the cover rate of robotic preparation up to 70% in routine practice for inpatients.

The reason for prolonged preparation time in robotic preparation seems to be reconstitution time for lyophilized drugs. The preparation time of CPA by ChemoRo® was reported to be 24 min, which is much longer than that of just a liquid drug, carboplatin (9.1 min) [14]. In our hospital, pre-reconstitution of lyophilized drugs, IFO, GEM, CPA, DXR, were started in January 2015. These drugs were selected by the confirmation of stability in aqueous solution over 24 h and by their high frequency of prescription. The average time for single preparation by robotics was gradually decreased after introducing the pre-reconstitution; these were 8.22 min, 7.54 min, 6.11 min in 2014, the first half of 2015 and the second half of 2015, respectively. In our routine practice, this pre-reconstitution procedure is usually performed after completion of daily preparations for patients (Fig. 3).

This study validated for the first time in a Japanese clinical environment the accuracy, safety, and feasibility of APOTECAchemo for the anticancer drug preparation. Indeed, APOTECAchemo demonstrated clinical feasibility for the application of liquid and lyophilized drugs, infusion bags from 50 mL to 500 mL, multiple sizes of syringe as final containers, and elastomeric pumps for continuous infusion. In addition, robotic preparation has a high traceability through sensors, photocells, a vision system, and barcode readers, providing all the most important information about compounding procedure, for example, picture image of used drugs, weight of ingredients, weight of solvent for reconstitution of lyophilized drugs and start and finish time of preparation. This traceability provides security for the operators of robotic preparation and for a patient safety concerns. Moreover, the high traceability enables the safe use of multi-dose vials in patient care settings, which may provide economical advantage for saving drug cost while reducing the quantity of disposal of residual hazardous drugs.

## Conclusion

The robotic preparation using APOTECAchemo has successfully implemented in Mie University Hospital and plays a prime role in the daily routine of anticancer drug compounding. The automated procedure yielded substantial advantages in drug compounding for accuracy, safety and feasibility.

## Abbreviations

BSC: Biological safety cabinet
CPA: Cyclophosphamide
DXR: Doxorubicin
FU: Fluorouracil
GEM: Gemcitabine
IFO: Ifosfamide
LoQ: Limit of quantification
NS: Normal saline
